# Supplementary material for: The Evolutionary Dynamics of the Mitochondrial tRNA in the Cichlid Fish Family
Source: Biology (Basel). 2022 Oct 18;11(10):1522. doi: 10.3390/biology11101522 (PMC9598224; doi:10.3390/biology11101522)
Supplement: Supplementary file 1 [file biology-11-01522-s001.zip › Supplementary Fig.pdf]

Supplementary Material

Supplementary Figures

A

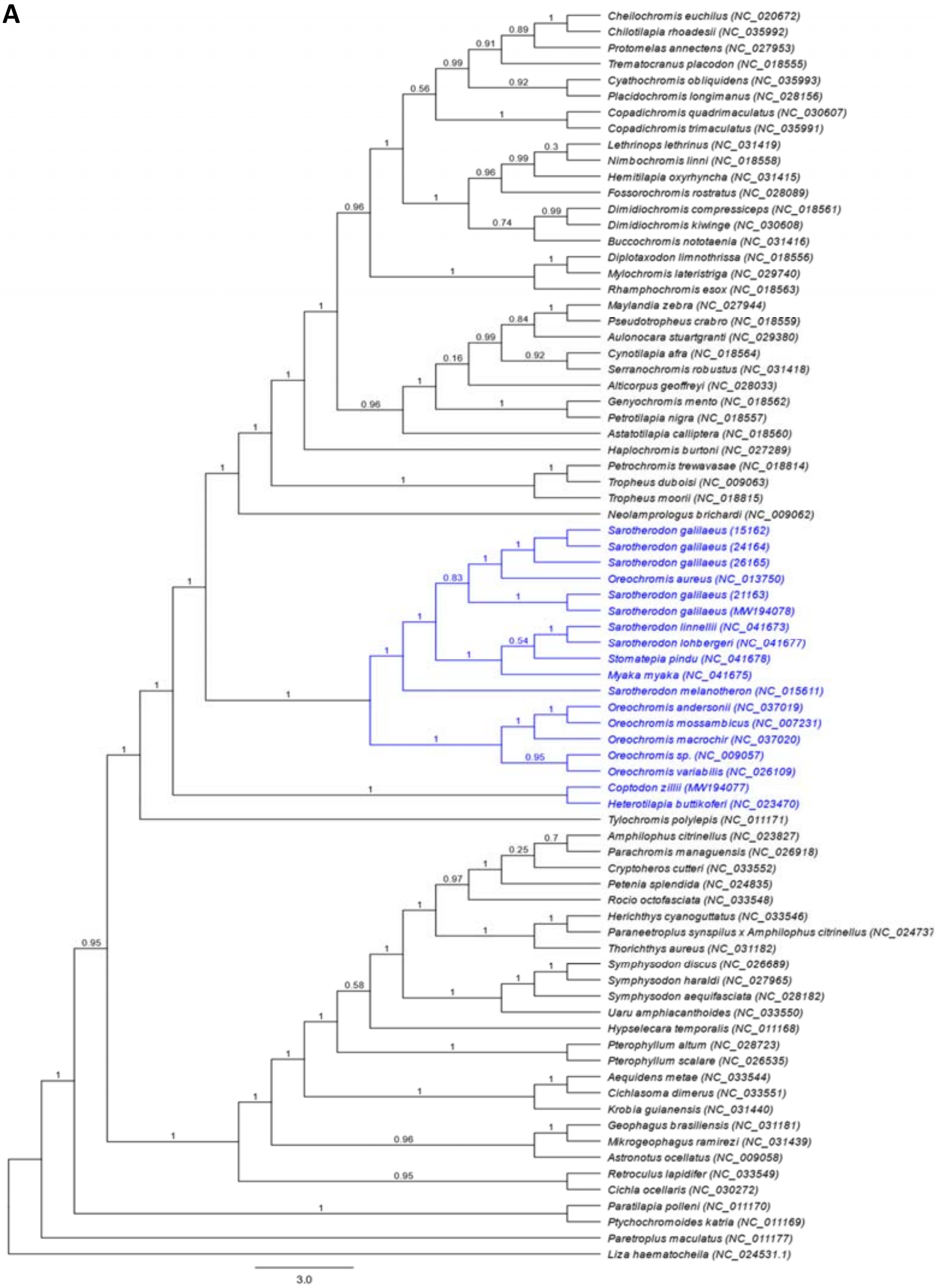

Supplementary Figure S1. The phylogenetic trees of cichlids species are inferred based on 78 mitogenomes from the GenBank database and rooted to *Liza haematocheila*. The tree constructs are based on complete mitochondrial genomes (A), tRNAs (B), CDS sequence (C), and IGS region (D) using the maximum likelihood method. The haplotilapiine lineage are together in the same clade, which is represented with the blue color.

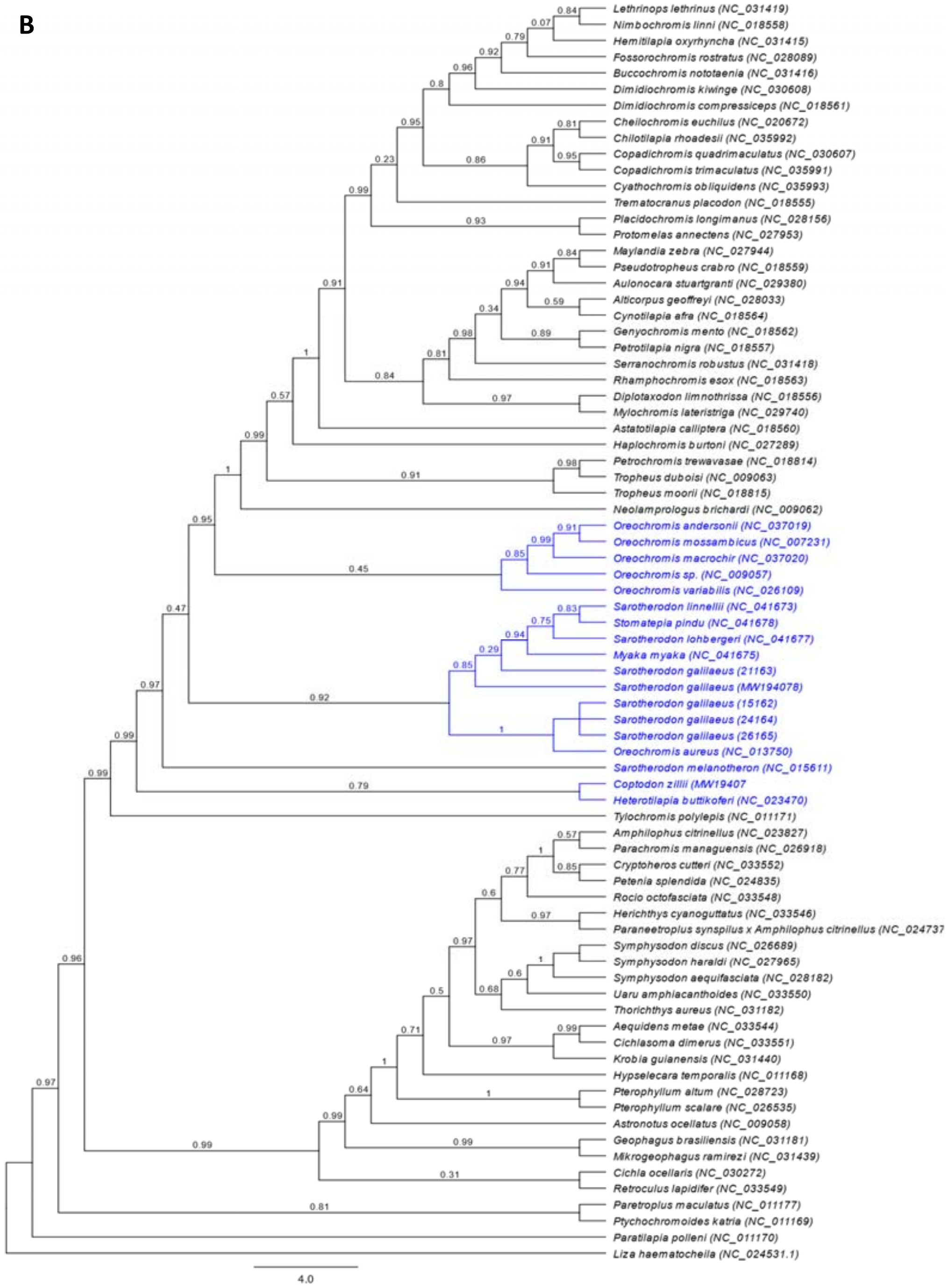

Supplementary Figure S1. Continue

C

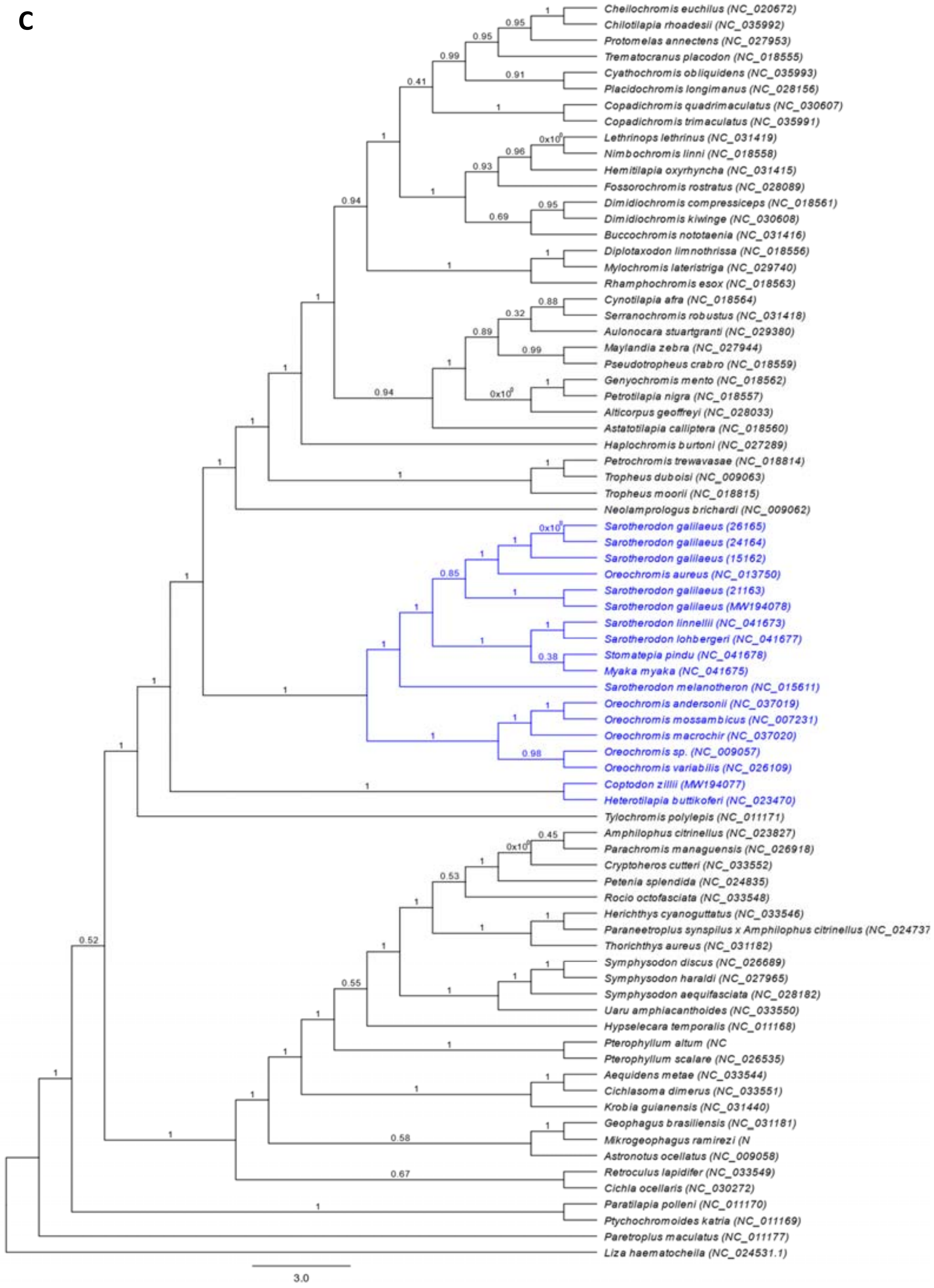

Supplementary Figure S1. Continue

D

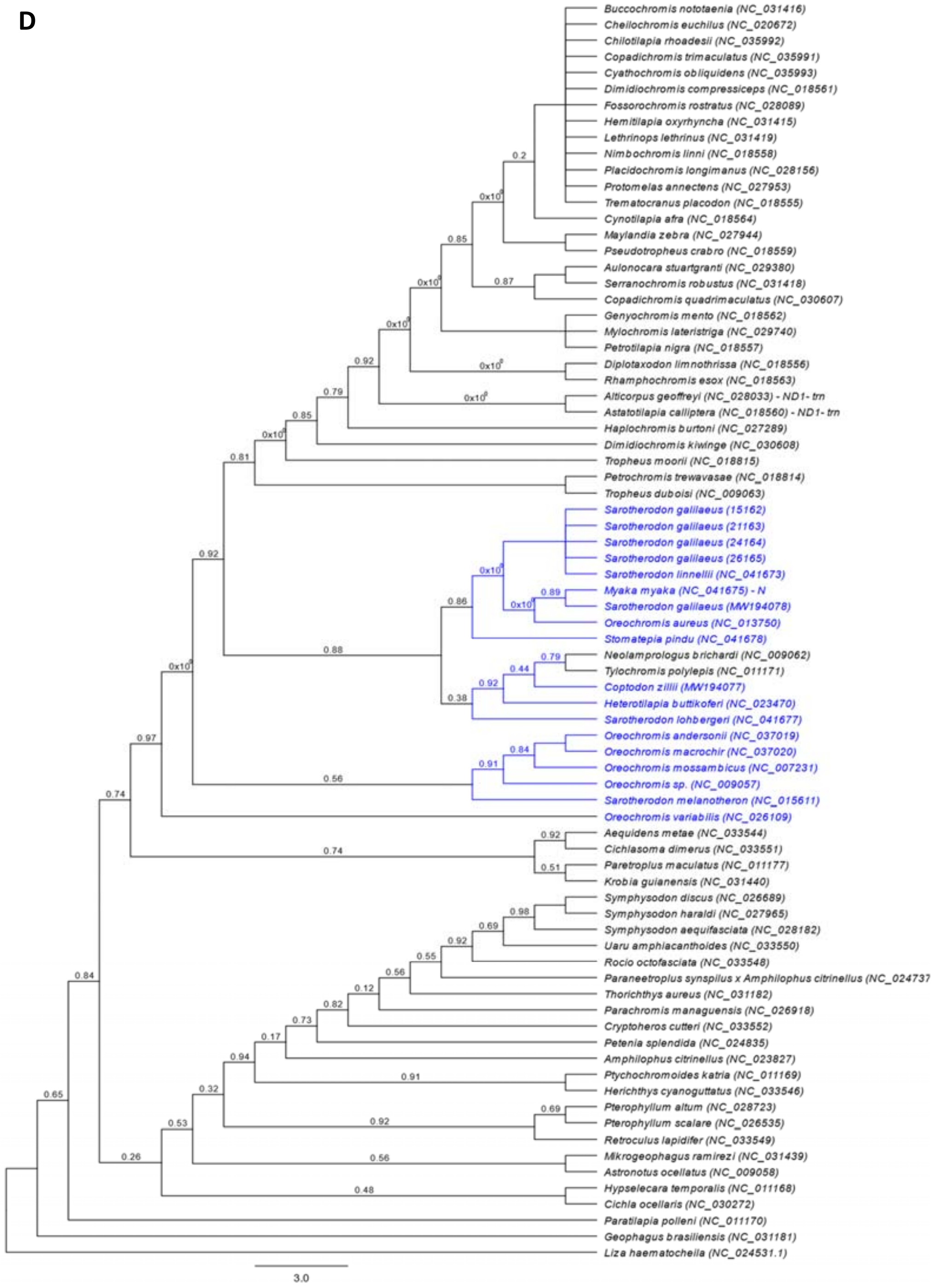

Supplementary Figure S1. Continue

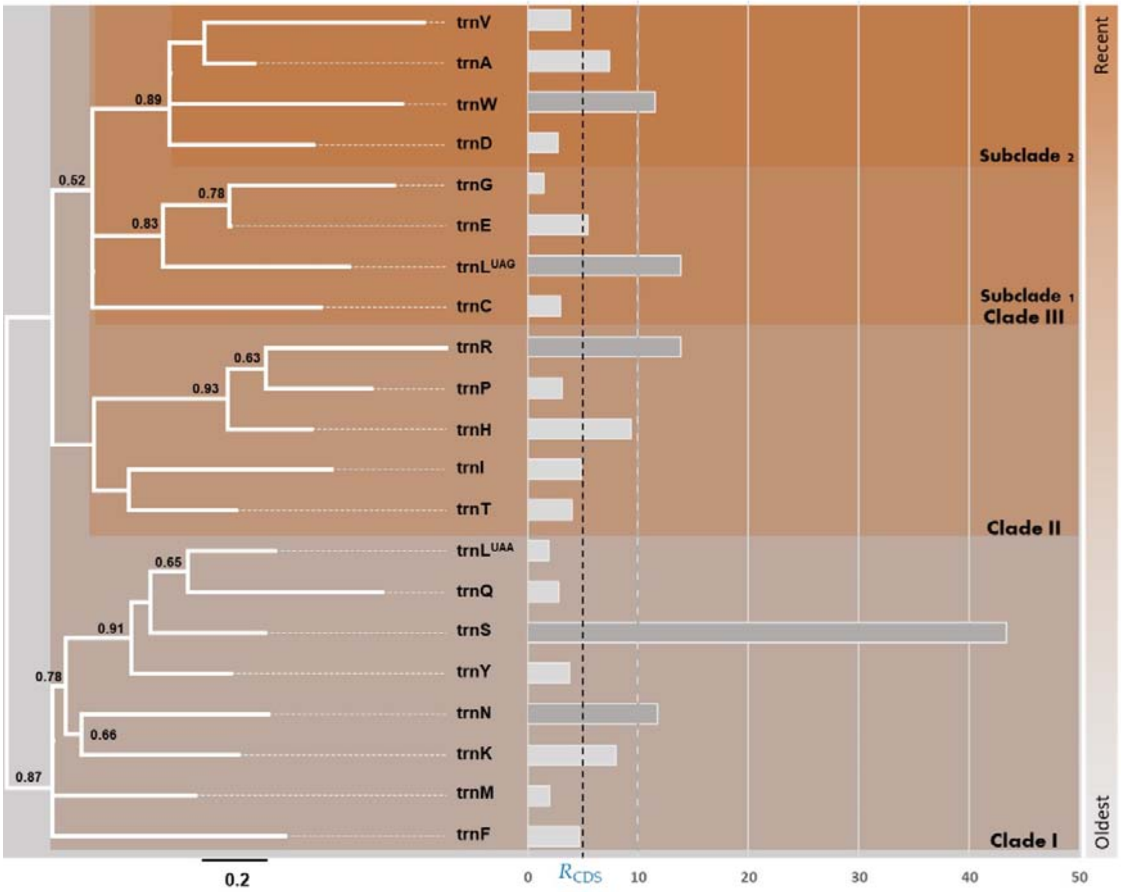

Supplementary Figure S2. The phylogenetic tree represents the evolutionary relationship among tRNA genes. The Histogram represents the R values of transition and transversion bias.

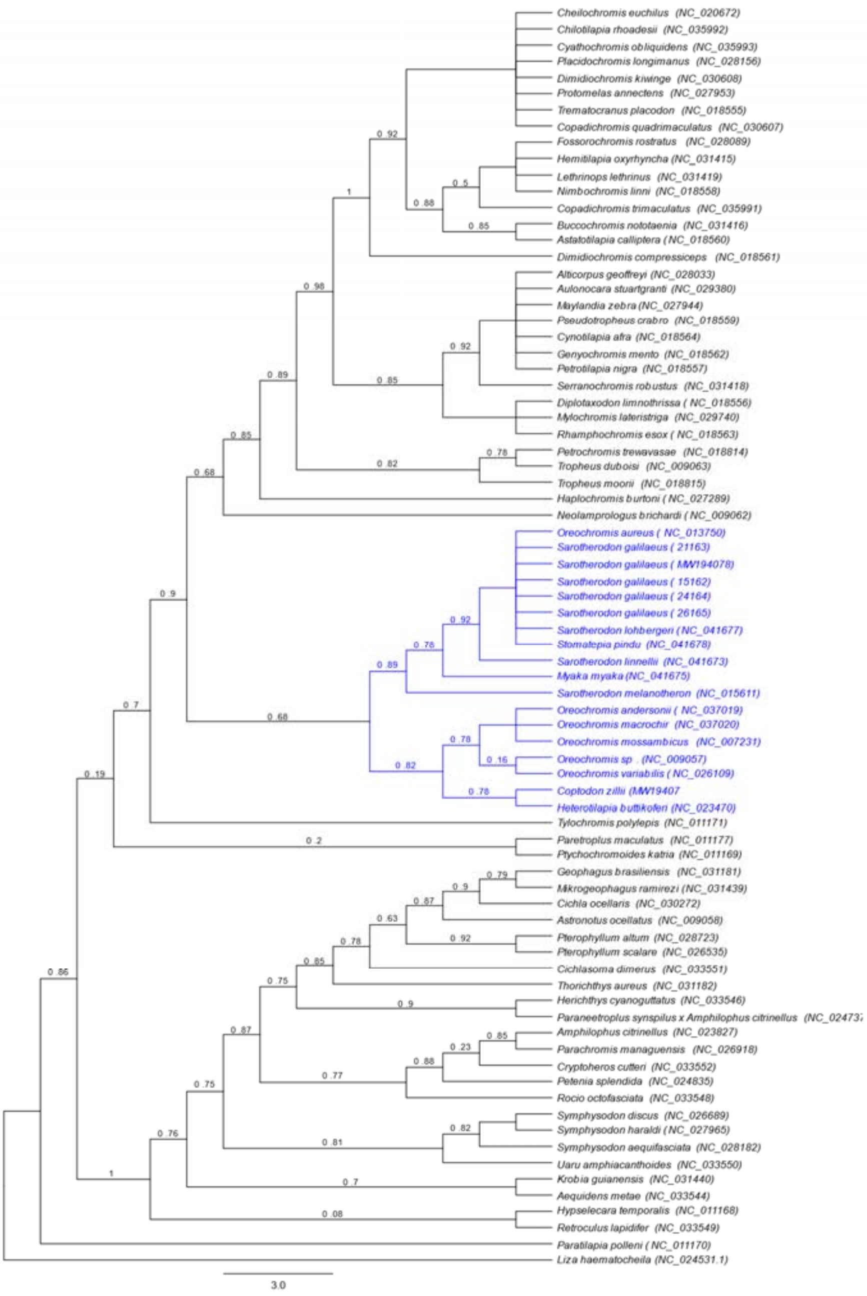

Supplementary Figure S3. The phylogenetic tree of cichlid species is inferred based on trnI and trnY genes sequences, using the maximum likelihood method and rooted to *Liza haematocheila*.

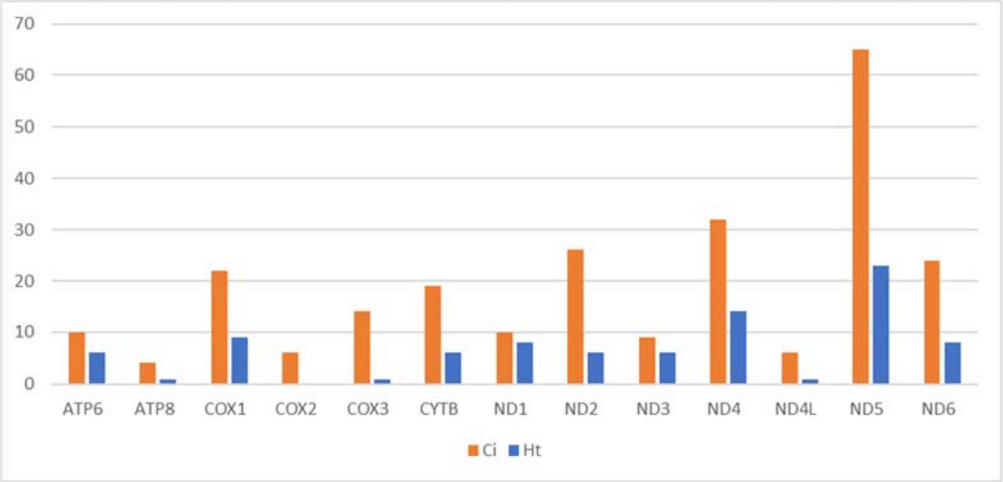

Supplementary Figure S4. The rate of mutations in coding DNA sequence (CDS) region. Both of ND5 and Nd4 record the highest rate of mutation in both cichlids and haplotilapiine lineage.
